# Failure of Recombinant Activated Factor VII in Treatment of Diffuse Alveolar Hemorrhage due to Cryoglobulinemic Vasculitis

**DOI:** 10.1155/2014/283086

**Published:** 2014-07-10

**Authors:** Dania Khoulani, Bharat Rao, Ammar Khanshour, Philip Kuriakose, Lenar Yessayan

**Affiliations:** ^1^Department of Internal Medicine, Henry Ford Hospital, 2799 W. Grand Boulevard, Detroit, MI 48202, USA; ^2^Department of Internal Medicine, Division of Hospital Medicine, Henry Ford Hospital, 2799 W. Grand Boulevard, Detroit, MI 48202, USA; ^3^Division of Hematology and Oncology, Henry Ford Hospital, 2799 W. Grand Boulevard, Detroit, MI 48202, USA; ^4^Division of Pulmonary and Critical Care Medicine, Henry Ford Hospital, 2799 W. Grand Boulevard, Detroit, MI 48202, USA; ^5^Division of Nephrology and Hypertension, Henry Ford Hospital, 2799 W. Grand Boulevard, Detroit, MI 48202, USA

## Abstract

Diffuse alveolar hemorrhage (DAH) is a serious complication of the small vessel vasculitis syndromes and carries a high mortality. Recombinant activated factor VII (rFVIIa) is used to treat bleeding in patients with hemophilia and antibodies to factor VIII or IX. It is increasingly being used in life-threatening hemorrhage in a variety of other settings in which conventional therapy is unsuccessful. Randomized controlled trials of rFVIIa in DAH are lacking. However, several case reports have described a complete or sustained control of DAH using rFVIIa after patients failed to respond to medical treatment. There are no case reports in the literature describing the use or the failure of rFVIIa in DAH associated with cryoglobulinemic vasculitis. We here report the failure of rFVIIa to control DAH in a patient with CD5+ B-cell non-Hodgkin's lymphoma and cryoglobulinemic vasculitis.

## 1. Introduction

Cryoglobulinemic vasculitis is a small vessel vasculitis that results from deposition of cryoglobulins on vessel walls and activation of the complement cascade. This will result in reactive damage in vessel walls causing tissue ischemia, necrosis, and bleeding [1, 2]. Pulmonary hemorrhage in cryoglobulinemic vasculitis carries a high mortality [3]. Several case reports and case series have reported successful control of diffuse alveolar hemorrhage in a variety of disease states and using either intravenous or intrapulmonary routes of administration [4–9]. We report the failure of intravenous rFVIIa to control DAH in a patient with cryoglobulinemic vasculitis.

## 2. Case Presentation

A 51-year-old female with a history of B-cell non-Hodgkin's lymphoma (NHL) with recent recurrence presented to the hospital with nausea, vomiting, and abdominal pain. She was diagnosed with CD5+ B-cell NHL on a submandibular mass biopsy 2 years prior to admission, which was treated with 6 cycles of R-CHOP, completing therapy 15 months prior to admission. Three months prior to admission, she noticed recurrent lymphadenopathy, which was being evaluated by oncology. She also then noticed a lower extremity rash, which was diagnosed as leukocytoclastic vasculitis on biopsy.

Laboratory findings on admission were as follows: hemoglobin 8.3 g/dL; leukocyte count 5,400/mm^3^; platelet count 52,000/mm^3^; serum creatinine 2.1 mg/dL; blood urea nitrogen 40 mg/dL from a baseline of 0.5 mg/dL and 20 mg/dL, respectively. The serum C3 level was 49 mg/dL (normal, 75 to 135) and C4 level was <2 mg/dL (normal, 13 to 75). Serological test for hepatitides B, C and HIV were negative. Rheumatoid factor was 71 IU/mL and DNA antibody titer was 80. She had strongly positive antinuclear antibodies and negative antineutrophil cytoplasmic antibodies. The thyroid stimulating hormone level was 203 mU/mL, free T4 0.27 ng/dL, and thyroid peroxidase antibody was positive. She was diagnosed with Hashimoto's thyroiditis and treated with intravenous levothyroxine. The test for cryoglobulin was positive for type 1 cryoglobulinemia (IgM kappa). The monoclonal IgM kappa protein level was 239 mg/dL. Urine protein was 114 mg/dL and urine creatinine 49 mg/dL. Urine microscopy revealed 0–2 red blood cells per high power field. Echocardiography indicated left ventricular diastolic dysfunction.

Lymph node biopsy on day 5 confirmed recurrence of her B-cell NHL. Renal biopsy on day 8 showed immune complex mediated proliferative glomerulonephritis suggestive of lupus nephritis and cryoglobulinemia. A bone marrow biopsy with aspirate (BMB-A) was planned on day 14; however, the patient left the hospital against medical advice on day 13. She did follow-up with oncology on day 14 and underwent a BMB-A, which showed bone marrow involvement with NHL.

She was rehospitalized on day 15 for shortness of breath and was treated for health-care acquired pneumonia with an 8-day course of vancomycin and aztreonam. Serial chest radiographs afterwards showed progressive worsening in bibasilar opacities interpreted at the time as worsening pulmonary edema. In light of her several autoimmune disorders in the setting of a low to moderate grade NHL, she was started on chemotherapy. She received 820 mg of intravenous rituximab (375 mg/m^2^) on day 30 and 197 mg of intravenous bendamustine (90 mg/m^2^) on day 31. On day 32 she developed respiratory distress and was rapidly intubated. Copious amounts of bloody tracheal secretions were noted. In light of this finding and the high serum cryoglobulin levels, she received 3 doses of methylprednisolone pulses (1 gram/day) followed by oral prednisone 1 mg/kg daily. Bronchoalveolar lavage showed progressively bloody lavage fluid over serial aliquots consistent with alveolar hemorrhage. Viral, fungal, and mycobacterial cultures were negative. Bacterial cultures grew* Stenotrophomonas maltophilia* that was treated with trimethoprim-sulfamethoxazole. She received daily plasmapheresis on days 33–39 and every other day thereafter. She remained severely hypoxemic and continued to have bloody secretions. On day 35, she was started on intravenous recombinant activated factor VII at 90 mcg/kg × 1 and then 60 mcg/kg every 6 hours for 3 doses. However, she continued to have copious bloody secretions, remained severely hypoxemic, and required periodic transfusions. On day 44, she received her second dose of intravenous rituximab (821.25 mg). Thereafter, her clinical condition continued to decline and the alveolar hemorrhage remained unabated. She was eventually terminally extubated on day 57 per the patient's family's request.

## 3. Discussion

We describe a patient with NHL, cryoglobulinemic vasculitis, and diffuse alveolar hemorrhage (DAH) who did not respond to conventional therapy. Her clinical presentation was consistent with DAH: dyspnea, hemoptysis, abnormal chest X-ray with bilateral alveolar infiltrates, and hypoxia. The diagnosis of DAH was confirmed bronchoscopically by lavage fluid that became progressively bloody over serial aliquots. The treatment of DAH depends on the underlying cause. Corticosteroids are a mainstay of therapy in most cases. Additional immunosuppressive therapy is dependent on the underlying disease (cyclophosphamide, azathioprine). Plasma exchange is recommended in the treatment of DAH associated with Goodpasture's syndrome, considered in refractory vasculitis syndromes, and in select cases of DAH associated with connective tissue disease. Drug-induced DAH and those related to exposures may be treated with withdrawal of the offending agent.

Recombinant activated factor VII (rFVIIa) is an approved coagulation factor for the treatment of patients with congenital or acquired hemophilia. Factor VII interacts with tissue factor (TF) found on deeper layers of vessel walls and the complex activates factor X leading to increased thrombin generation which in turn coverts fibrinogen to fibrin and results in clot formation. In high doses, rFVIIa can activate factor X in the absence of TF. There is a growing literature supporting the use of rFVIIa to stop active pulmonary hemorrhage in DAH. Intrapulmonary and systemic rFVIIa administrations in DAH have been described. Anecdotal reports of successful use in patients with DAH have been described following a variety of insults, including microscopic polyangiitis [4–6], allogeneic stem cell transplantation [7], leukemia [5, 8], and systemic lupus erythematosus [6]. Doses that have been successful include intrapulmonary administration of 50 mcg/kg rFVIIa in 50 mL of sodium chloride by bronchoalveolar lavage with 25 mL in each of the bronchi [8, 9]. Intravenous regimens include 90 mcg/kg every 4 hours × 3 doses and repeated upon recurrent hemorrhage days later [10] and 90 mcg/kg in a single dose [5] and 120 mcg/kg every 3 hours × 3 doses [6]. DAH in the described case reports resolved with rFVIIa therapy; however, there were two case reports in which the patients died of their underlying disorder [7]. In one of the cases described in the literature, the patient's acute episode of DAH resolved with treatment, but he later died of recurrent pulmonary hemorrhage after he was discharged [8].

Pulmonary hemorrhage in cryoglobulinemic vasculitis carries a high mortality rate [3]. It occurs in less than 5% of patients with mixed cryoglobulinemia and is generally associated with cryoglobulinemic glomerulonephritis. Our patient had widespread vasculitis, which is defined as involvement of the skin and at least two other organs. She had skin and kidney involvement confirmed by biopsy, and lung involvement confirmed bronchoscopically by lavage fluid that became progressively bloody over serial aliquots. She failed standard treatment including several plasma exchange sessions, methylprednisolone therapy, and rituximab therapy. She also failed intravenous rFVIIa therapy at 90 mcg/kg × 1 and then 60 mcg/kg every 6 hours × 3 doses. Her failure to respond to rFVIIa may be due to high levels of cryoglobulins impeding interaction between rFVIIa and tissue factor as a result of her widespread cryoglobulinemic vasculitis. There is also a possibility that her mild thrombocytopenia may have contributed to her failure to respond to rFVIIa. Finally, we administered rFVIIa systemically. Some have suggested that pulmonary hemostasis can be induced more effectively from the alveolar side in DAH than from the endothelial side. This view point is supported by abundant intra-alveolar expression of TF in inflammatory pulmonary conditions [11–13]. In a case series of 6 patients, Heslet et al. reported sustained hemostasis in all patients following either a single dose of rFVIIa or repeated rFVIIa administration [9].

In conclusion, although both systemic and intrapulmonary rFVIIa administration have been reported to achieve pulmonary homeostasis in several case reports and case series, randomized controlled trials are necessary to establish the efficacy of rFVIIa in various disease states associated with DAH.
